# Digital Phenotyping in Mental Health - What can it mean for the future of Psychiatry?

**DOI:** 10.1192/j.eurpsy.2025.477

**Published:** 2025-08-26

**Authors:** M. J. Brito, J. D. Vieira-Andrade

**Affiliations:** 1 Serviço de Psiquiatria, Unidade Local de Saúde de Coimbra; 2 Instituto de Psicologia Médica, Universidade de Coimbra, Coimbra, Portugal

## Abstract

**Introduction:**

Smartphones, central to modern life, offer a cost-effective tool for gaining patient insights outside the consultation room. Through passive data collection (e.g., sensor data) and active questioning, smartphones enable ecological assessments of psychiatric symptoms and self-reported experiences. This “moment-by-moment quantification of the individual-level human phenotype in situ using data from personal digital devices” via digital phenotyping (DP) has garnered significant research attention, showing potential for early detection and intervention in mental health.

**Objectives:**

We explore recent DP developments in mental health, highlighting its potential to transform clinical practice while acknowledging challenges and risks.

**Methods:**

Narrative literature review resorting to PubMed and Google Scholar using keywords such as “digital phenotyping”, “digital phenotype”, “digital biomarker” and “mobile sensing”.

**Results:**

DP studies, particularly in Mood Disorders and Schizophrenia, mostly rely machine learning for data analysis. Biomarkers from passive data (e.g., GPS, social connectivity, physical activity) correlate with self-reports and clinical measures of depression, anxiety, mania, and psychosis. Speech and text analysis through Natural Language Processing (NLP) offers new research avenues. DP promises early detection, relapse prevention, and treatment monitoring but faces challenges, including privacy concerns, and low user engagement - that could be solved by closing the loop by returning individual research results or a tailor-made intervention. Nevertheless, regulation and good practice standards are still lacking, posing the threat of diagnostic inaccuracy and undeniable iatrogenic risk.

**Conclusions:**

For DP to fully realize its potential, integration with standard care and existing systems is essential. While risks exist, when comparing DP with other medical interventions currently under research, perils are minor. Mental health care urgently needs disruptive innovation to improve access and quality.

**Disclosure of Interest:**

None Declared

